# Vaccination of Dogs

**Published:** 1856-03

**Authors:** 


*Vaccination of Dogs.*—We find in the Deutsche Klinic, a suggestion which may be of value to the lovers of dogs. In number seven of that journal for 1855, Dr. Sautlus states that he is acquainted with a sportsman enjoying some reputation as a trainer of dogs, who vaccinates all the pups on the nose. He contends that this operation entirely protects the animal from the distemper.

